# Spatial mapping and predictors of ever-tested for HIV in adolescent girls and young women in Ethiopia

**DOI:** 10.3389/fpubh.2024.1337354

**Published:** 2024-04-03

**Authors:** Mulugeta Shegaze Shimbre, Abayneh Tunja, Belay Boda Bodicha, Abebe Gedefaw Belete, Samuel Hailgebereal, Lovel Fornah, Wei Ma

**Affiliations:** ^1^Department of Epidemiology, School of Public Health, Cheeloo College of Medicine, Shandong University, Jinan, Shandong, China; ^2^Schools of Public Health, College of Medicine and Health Sciences, Arba Minch University, Arba Minch, Ethiopia; ^3^Department of Biomedical Sciences, School of Medicine, College of Medicine and Health Sciences, Arba Minch University, Arba Minch, Ethiopia; ^4^School of Public Health, College of Medicine and Health Sciences, Wachamo University, Hosaena, Ethiopia

**Keywords:** mapping, ever tested for HIV, adolescent girls, young women, Ethiopia

## Abstract

**Background:**

Adolescent girls and young women (AGYW) are expected to be healthy in life. However, the unique health challenges faced by AGYW include unsafe sex practices and substance abuse. Only 46.3% of AGYW in Africa are aware of their HIV status, and difficulties are underlined in HIV testing among adolescents and young people. To demarcate the areas with low and high HIV testing, this study aimed to map predictors of ever-tested for HIV among adolescent girls and young women in Ethiopia.

**Methods:**

Secondary data analysis was conducted using the dataset from the 2016 Ethiopia Demographic and Health Survey (EHDS). We conducted spatial autocorrelation and Moran's I statistics to investigate the regional variance of HIV being ever-tested in AGYW. In addition, spatial regression analyses such as ordinary least squares (OLS) regression and geographically weighted regression (GWR) were carried out to determine the predictors of being ever-tested for HIV among AGYW.

**Results:**

Addis Ababa, some parts of Amhara, Dire Dawa, Gambela, and Tigray were the primary regions and city administrations for being ever-tested for HIV among AGYW. A lesser proportion of AGYW being ever-tested for HIV was found in Somalia, Afar, Benshangul Gumuz, and southern nations. Spatial regression analyses identified an age range of 15–19 years, being Muslim, having no formal education, having no knowledge about HIV, and experiencing severe stigma as predictors of being ever-tested for HIV among AGYW.

**Conclusion:**

The proportion of AGYW being ever-tested for HIV was high in Addis Ababa, some parts of Amhara, Dire Dawa, Gambela, and Tigray. Spatial regression analyses identified that AGYW aged 15–19 years, having no formal education, having no knowledge about HIV, and experiencing severe community stigma as predictors negatively affecting the proportion of being ever-tested for HIV, while being Muslim was a predictor that positively affected the proportion of being ever-tested for HIV. The governments and other stakeholders should focus on increasing HIV testing among these special groups of the population.

## Introduction

The HIV epidemic continues to spread globally, though at varying rates across regions and countries. In Sub-Saharan Africa (SSA), adolescent girls and young women (AGYW) are at a notable risk of acquiring HIV, with nearly 63% of all newly reported HIV infections in 2021 occurring within this demographic population (1). Youth constitute an increasing proportion of people with HIV/AIDS, specifically AGYW, with a concentration of over 2.8 million people under the age of 19 with HIV in 2020 in eastern and southern Africa (2, 3). Globally, more than two-thirds of new HIV infections are found in African nations, which are home to 60% of youth under the age of 25 years (2). In Sub-Saharan Africa, the proportion of HIV infection is high and widespread, with South Africa accounting for the majority (3). Over two-thirds of the world's youth living with HIV are found in this region (2).

Research conducted across seven African nations revealed that 3.6% of individuals in this demographic were living with HIV (4). A systematic review and meta-analysis across 10 high-prevalence African countries found variations in HIV incidence rates among different geographical locations and age groups (5). In 2019, there was an expected decrease in the population of adolescents and youth living with HIV globally, particularly in Eastern and Southern Africa (6). However, the reduction in new infections may not be rapid enough to eliminate AIDS as a public health threat among individuals of this age group. This demographic population tends to seroconvert earlier than their male counterparts and constitutes ~30% of all new cases (7). Risk factors such as a lack of family support and inadequate education are associated with a high HIV incidence among adolescent girls and young women (AGYW) (8). Furthermore, insufficient awareness of HIV prevention measures, including HIV testing, contributes to the high HIV incidence among AGYW (4). For example, only 46.3% of AGYW are aware of their HIV status (4). A study also highlighted the challenges faced in HIV testing among adolescents and youth (9).

Studies conducted in Ethiopia reveal a higher prevalence of HIV among AGYW compared to men of the same age group and older women, especially among the highly vulnerable groups (10). Despite this high proportion, the uptake of HIV testing among AGYW remains low, with only 21.8% reporting testing in the past year. However, people living with HIV and those receiving antiretroviral therapy tend to engage in safer sexual behaviors (11). In Sub-Saharan African countries, including Ethiopia, HIV testing uptake among AGYW is low but is showing an increasing trend (6, 12, 13). Factors associated with a higher uptake of HIV testing include older age (14), better education, comprehensive HIV knowledge (15), discussions about HIV with mothers or female guardians (14), having multiple sexual partners, having nondiscriminatory attitudes, and pregnancy (12), whereas factors such as being unmarried, living in rural areas, having lower financial status, having limited media exposure, experiencing early sexual initiation, having perceived low risk, and having only one sexual partner are associated with lower HIV testing uptake (13, 15).

In Ethiopia, HIV prevalence is higher among urban and young populations, with an estimated 1.52% prevalence among women (16). A smaller proportion of AGYW (27%) tested for HIV (17). These figures also vary from region to region (11, 16–18). For an AIDS-free generation and the pandemic to be under control, prevention of HIV infection in this age group is essential (7). To manage the pandemic among AGYW, the government must make consistent efforts to detect HIV infection in such individuals (4).

GPS technology helps integrate spatial data, including demographics, location of healthcare facilities, HIV prevalence, and critical areas requiring targeted testing strategies (19). However, the spatial distribution of HIV being ever-tested AGYW has not been examined in Ethiopia. Therefore, this study aimed to identify hot spots and cold spots for HIV being ever-tested in AGYW to enhance testing strategies and improve the response to HIV/AIDS (11, 16–18).

## Methods

### Study area and population

The current study was conducted in Ethiopia, the second-most populated nation in Africa located in the Horn of Africa between 33 and 48°E longitude and 3° and 15°N latitude. The Ethiopian Public Health Institute (EPHI) conducted a comprehensive study on the demographic and health characteristics of the nation, as commissioned by the Ethiopia Federal Ministry of Health. Ethiopia is geographically partitioned into nine distinct regions. The 2016 Ethiopia Demographic and Health Survey (EDHS) was stratified, and the sample was drawn using a two-stage process. In the first phase, a sample of 645 enumeration areas (EAs) was chosen, consisting of 202 EAs in urban areas and 443 EAs in rural areas. The selection procedure was implemented by using a probability proportional to the size of each EA taken from the sample frame of the Population and Housing Census (PHC) in 2007. In the second stage, 28 households were selected in each cluster. The EDHS conducted in 2016 encompasses a comprehensive representation of all geographical areas and municipal governments (20). The specifics of sampling techniques for EDHS populations are covered elsewhere (20).

**Figure 1:**
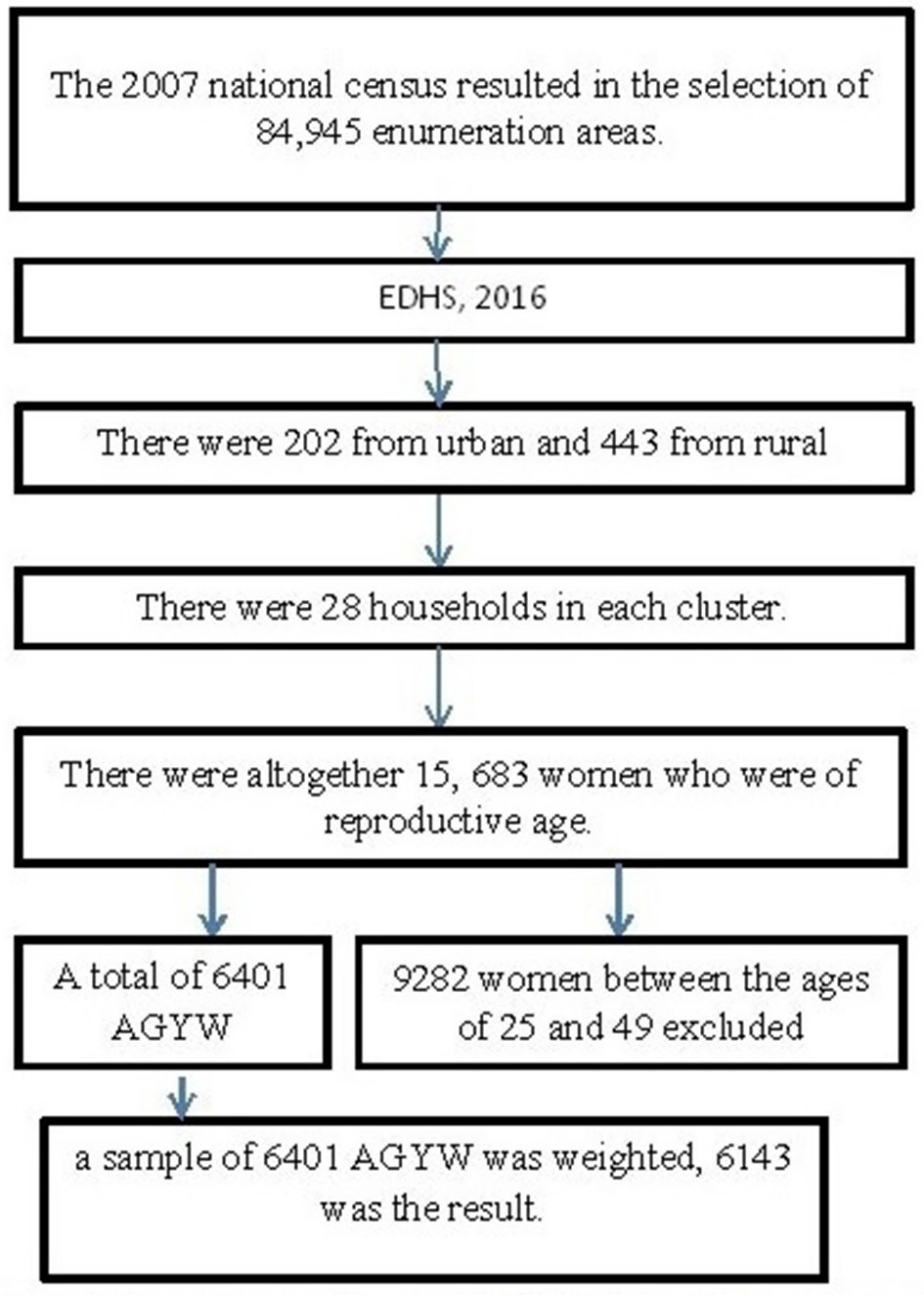
Sampling frame, sampling procedure, and data extraction process. AGYW, adolescent girls and young women; EHDs, Ethiopia Demographic and Health Survey.

As part of the EDHS 2016, all women aged between 15 and 59 years were assessed for ever having an HIV test (Figure 1). AGYW aged 15–24 years were included in the analysis, while women aged >24 years were excluded from this study.

### Outcome and explanatory variables

#### Dependent variable

The dependent variable was being ever-tested for HIV and coded as “no = 0” or “yes = 1”. The proportion of individuals who have ever been tested for HIV was calculated. The number of respondents who answered “yes” was divided by the total number of both “yes” and “no” responses for being ever-tested for HIV.

#### Explanatory variables

The variables related to HIV testing status were selected based on an in-depth analysis of the existing literature (21, 22). The variables measured in this research include age, education attainment, employment status, marital status, place of residence, wealth index, religious affiliation, knowledge of HIV, awareness of sexually transmitted infections (STIs), number of sexual partners, exposure to media, stigma, geographical region, and proximity to healthcare facilities. In this study, all of these variables were taken into account for spatial regression analysis.

### Operational definitions and measurement

#### Knowledge about HIV

This variable evaluated the participant's knowledge about HIV based on the combination of eleven knowledge indicator questions: (1) physically healthy-looking individuals can have HIV; (2) the risk of acquiring HIV can be minimized by having one sexual partner; (3) HIV transmitted through mosquito bites; (4) sharing food with an AIDS patient can transmit HIV; (5) HIV can be transmitted through supernatural means or witchcraft; (6) the risk of getting HIV can be increased by never skipping the condom during sex; (7) during pregnancy, HIV can be transmitted; (8) during delivery, HIV can be transmitted; (9) breastfeeding can transmit HIV; (10) if a husband or partner has sexually transmitted infection, the spouse reasonably asking the husband to use a condom; and (11) during pregnancy, there are drugs to be used to avoid HIV transmission to the baby. Participants' knowledge of HIV was categorized into not knowledgeable (scored ≤ 5) and knowledgeable (score 6–11) based on the right response to all of the indicators (23).

#### Access to health facility

Participants were assessed for accessibility to the health facility. If the facility was not far from the residence, the participant responded by citing it as no big problem, while if it was far from the residence, the participant responded by citing it as a big problem.

#### Exposure to media

Based on their prior history, the respondents were assessed for exposure to reading magazines or newspapers, listening radio, watching television, or using the internet to generate a variable (exposure to media). The response was coded as “0 = no” if an individual had no access to any of the media listed above and “1 = yes” if an individual had access to at least one of these media. The proportion of exposure to media was generated using “yes” as the numerator and “yes” plus “no” as the denominator.

#### Risky sexual activities

AGYW were assessed for at what age sex was started and multisexual partners (23). The age when first sex was started and the proportion of multisexual partners were also generated.

#### Community stigma

Seven measures that show unfavorable views toward people living with HIV were used to generate community stigma as follows: (1) children who acquired HIV should not be allowed schooling with children who did not acquire HIV; (2) people would not purchase fresh vegetables from vendors who are living with HIV; (3) people lose respect for those who have HIV or who are suspected of having it; (4) people speak negatively about such individuals; (5) because of how others might respond, people are reluctant to get an HIV test; (6) if a member of the family has HIV, it would cause them to be embarrassed; and (7) people fear of contracting HIV from touch with a contaminated person's saliva. This index was then classified as having no stigma if the subject scored 7, low stigma if the participant scored 6, moderate stigma if the participant scored (4, or 3), and high stigma if the individual scored 2 or 1 (23). Then, the proportion was generated for each category.

#### Wealth index

The proportions of AGYW in the wealth quintile, namely, poorest, poor, middle, rich, and richest, were generated from data on the combined wealth index. The household wealth index scores were calculated by summing the weighted scores of the indicators of households such as televisions and bicycles, materials used for housing construction, and types of water access and sanitation facilities.

### Data extraction and management

The EDHS 2016 data were obtained from the DHS website after a formal online request for data. The request included a comprehensive explanation of the objectives of the research. The data are accessible for downloading on the official website of the DHS:http://www.dhsprogram.com. We downloaded the data from the DHS website and used the ETIR71DT folder to identify and extract important information for the research investigation. We also formally requested and downloaded the geographical coordinates such as latitude and longitude to visualize the hotspot areas. To maintain the confidentiality of the participants, the geographic coordinates have been deliberately displaced by a distance of 5 km in a random manner. We used Stata SE 18 (STATA Corporation. IC., TX, USA) (24) to extract variables from the original study dataset, clean, and conduct descriptive analysis.

### Secondary data analysis

The global Moran's I statistic was analyzed to investigate the presence of spatial autocorrelations. A geographical autocorrelation was assessed using the global Moran's I statistic to determine the spatial pattern of HIV testing across different regions in Ethiopia. The spatial autocorrelation can show patterns of dispersion, clustering, or random distribution of outcome measures throughout the study areas (25). Global Moran's I is a widely used geographic statistical measure that evaluates the presence of spatial autocorrelation. It has a single output value that ranges from −1 to 1. A Moran's I value of −1 implies that the distribution of individuals who have been tested for HIV is low. In contrast, a Moran's I value of +1 implies a spatial clustering of being ever-tested for HIV. A Moran's I value of 0 indicates that the distribution of individuals who have ever been tested for HIV is random. The spatial autocorrelation patterns within the study area were analyzed using the Getis-Ord Gi^*^ statistics. The realization of this outcome was accomplished by the computation of the GI^*^ statistic for every region. The statistical significance cluster is determined using a *p*-value of >0.05. The statistically significant findings imply that a high geographic information (GI) value is suggestive of “hotspots” defined by an increased likelihood of HIV testing, whereas places with a low GI value are related to “cold spots,” which imply a reduced probability of HIV testing (26).

Spatial regression is a statistical methodology used to examine the association between a dependent variable and independent variables. The spatial regression analysis method could involve both local and global analysis techniques (27). To ensure the coefficient variability within each cluster or region, we first examined the global geographical regression models and then performed local geographical analysis (25).

Following that analysis, we conducted OLS regression with the appropriate tests to verify the assumptions. The normality assumption of the residuals was evaluated using the Jarque–Bera test. To assess the presence of spatial autocorrelation in the geographically weighted regression model, the Koenker BP test was used. The use of this test was justified due to the observation of non-spatial autocorrelation in the residuals. To reduce redundancy among the independent variables, the presence of multicollinearity was evaluated using the variance inflation factor (VIF) to confirm its value was below a certain threshold of 10 (28–30).

The geographically weighted regression (GWR) analysis was conducted using ArcGIS 10.8 software. The criteria were used to develop a spatial regression model including the coefficients that satisfy the previously specified criteria: they demonstrate the anticipated directionality, there is no presence of multicollinearity among the explanatory variables, the coefficients are statistically significant, and they display high adjusted *R*^2^ values. Variable coefficients showed that a *p*-value of <0.05 was selected. The most appropriate model for the data was selected by considering the lowest AICc score and a higher adjusted *R*^2^ value (28–30).

### Ethical statement

This secondary data analysis did not involve participants' contacts. Thus, informed consent is not required in this study. The uses of the secondary data were formally requested for the current study, with permission being granted to identify the research questions and objectives, conduct analysis, and develop a manuscript. The original study also collected data according to the Declaration of Helsinki. The DHS dataset does not include personal identifiers such as names of people. Furthermore, the data were handled with confidentiality, and it is imperative to refrain from any endeavor to ascertain the identity of any household or individual respondent who participated in the survey. The information obtained was only used for statistical reporting and analysis of our registered study.

## Results

### Background, knowledge, and behavior characteristics of AGYW in the study

A total of 6,143 AGYW aged 15–24 years were included in this study. More than half of the responders (55%) were aged 15–19 years. Of the total AGYW, only 25.7% attained secondary or higher education. A majority of the respondents (57%) were never married, and 37.4% of the responses from respondents were coded as missing values. In terms of religion, 43% of the respondents identified as orthodox. Rural inhabitants made up more than two-thirds (73.1%) of the respondents, and 33% of AGYW were from low-income families. Regarding media exposure, approximately half of them (50.8%) were not exposed to any newspapers, radio, television, or internet, while 26.3% of AGYW had no knowledge about HIV. Regarding the testing place, 25.3% of AGYW did not know the place of HIV testing, and 48.3% of them mentioned that distance to health facilities was a big problem (Table 1).

**Table 1:** Background, knowledge, and behavioral characteristics of AGYW in Ethiopia.

**Variable**		**Frequency**	**Percentage**
Age of AGYW in years	15–19 years	3,381	55.04
	20–25 years	2,762	44.96
Education attainment	No formal education	1,230	20.03
	Primary education	3,333	54.25
	Secondary and above	1,580	25.72
Marital status	Not ever been married	3,500	56.97
	Married (formally and cohabited)	346	8.99
	Recorded as the missing values	2,297	37.40
Religion	Orthodox	2,640	42.97
	Muslim	1.883	30.65
	Others	1.621	26.38
Residence	Urban	1,467	23.89
	Rural	4,676	73.11
Wealth index	Poorest	945	15.39
	Poor	1,080	17.59
	Middle	1,114	18.13
	Rich	1,229	20.01
	Riches	1,774	20.88
Media exposure	No media exposure	3,025	49.24
	Had exposure to media	3,118	50.76
Knowledge of HIV	Not knowledgeable	1,638	26.67
	Knowledgeable	4,505	73.33
Distance to healthcare facilities	Was not problem	3,174	51.67
	Was a big problem	2,969	48.33
Knowing the HIV testing place	No	1,552	25.26
	Yes	4,198	68.35
	Recorded as the missing values	393	6.39
Age at first sex in a year	Had no sex	3,278	53.36
	8–15	564	9.18
	15–18	1,789	29.13
	19–23	512	8.33
Sexual partner, excluding spouse	No	5,968	97.16
	1 or more	175	2.84
Community stigma	No stigma	44	0.72
	Low stigma	2,562	49.07
	Moderate stigma	2,400	34.73
	Severe stigma	1,136	7.69

In terms of sexual exposure, 53.4% of adolescent girls and young women (AGYW) reported no sexual activity, while 9.2 and 29.1% had initiated sex between the ages of 8–14 and 15–19 years, respectively. Regarding sexual partners, the majority of AGYW (97.2%) reported no sexual activity, excluding spouses. Among all participants, 41.7% and 18.5% reported low and severe community stigma related to HIV, respectively (Table 1).

### Spatial autocorrelation and HIV testing clusters

**Figure 2:**
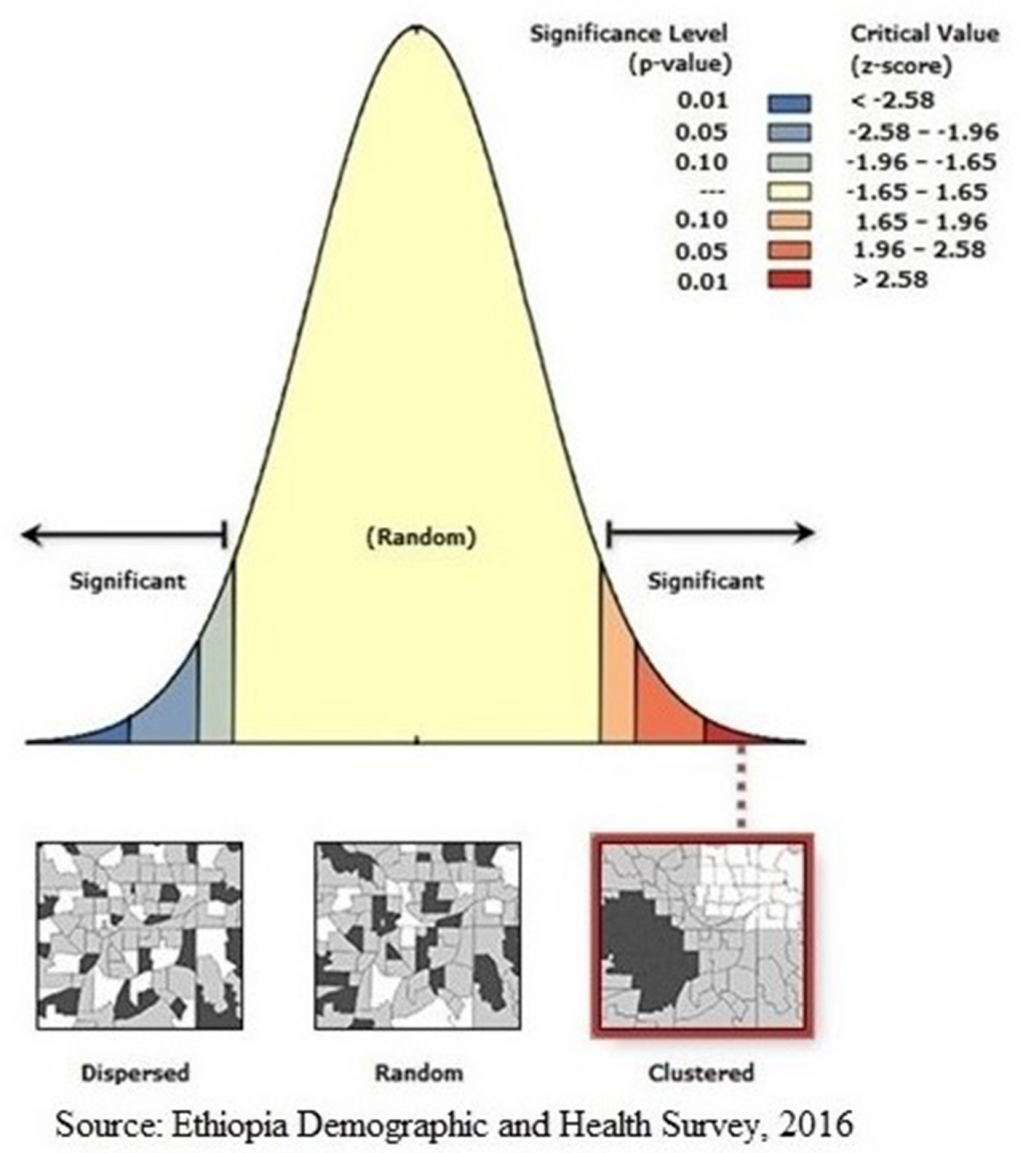
Global Moran's I analysis of AGYW being ever-tested for HIV. The Moran's I index value indicated that ever-tested. The Moran's I index value indicated that ever-tested pattern is not random. The significant *P*-value and positive *z*-score showed the spatial distribution of high values and low values in this study was more spatially clustered, while significant *P*-value and negative *z*-score showed the spatial distribution of high values and low values in this study was more spatially dispersed.

**Figure 3:**
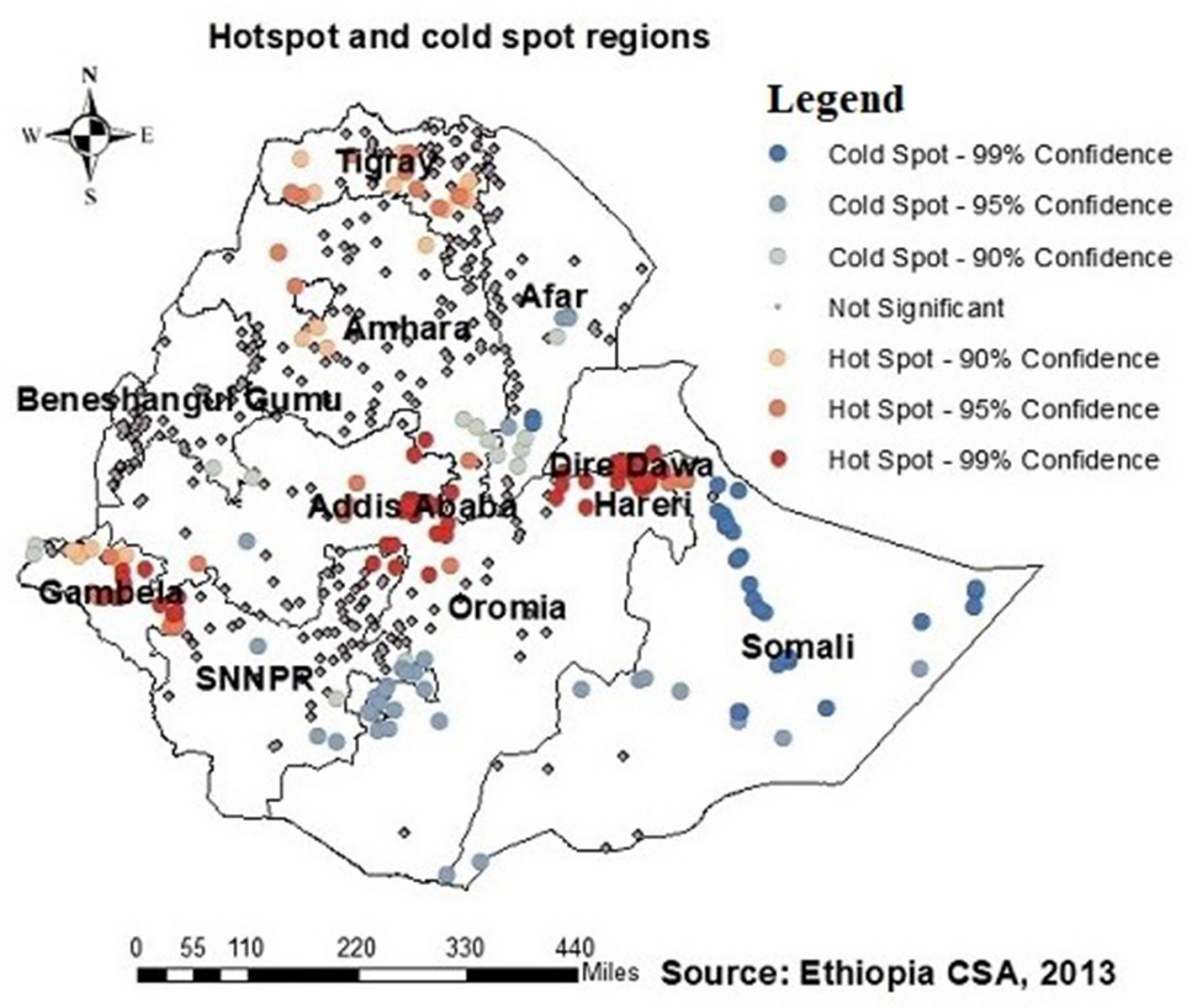
Hotspots and cold spots of AGYW being ever-tested for HIV in Ethiopia. Hotsport regions (red colors) were Addis Ababa, Northern Amhara, Dire Dawa, Tigray, and Gambella, while cold spot regions (blue colors) were Afar, most of Mahara, Somalia, Benishangul-Gumuz, and Southern Nation, Nationality and People Region (SNNPR).

**Figure 4:**
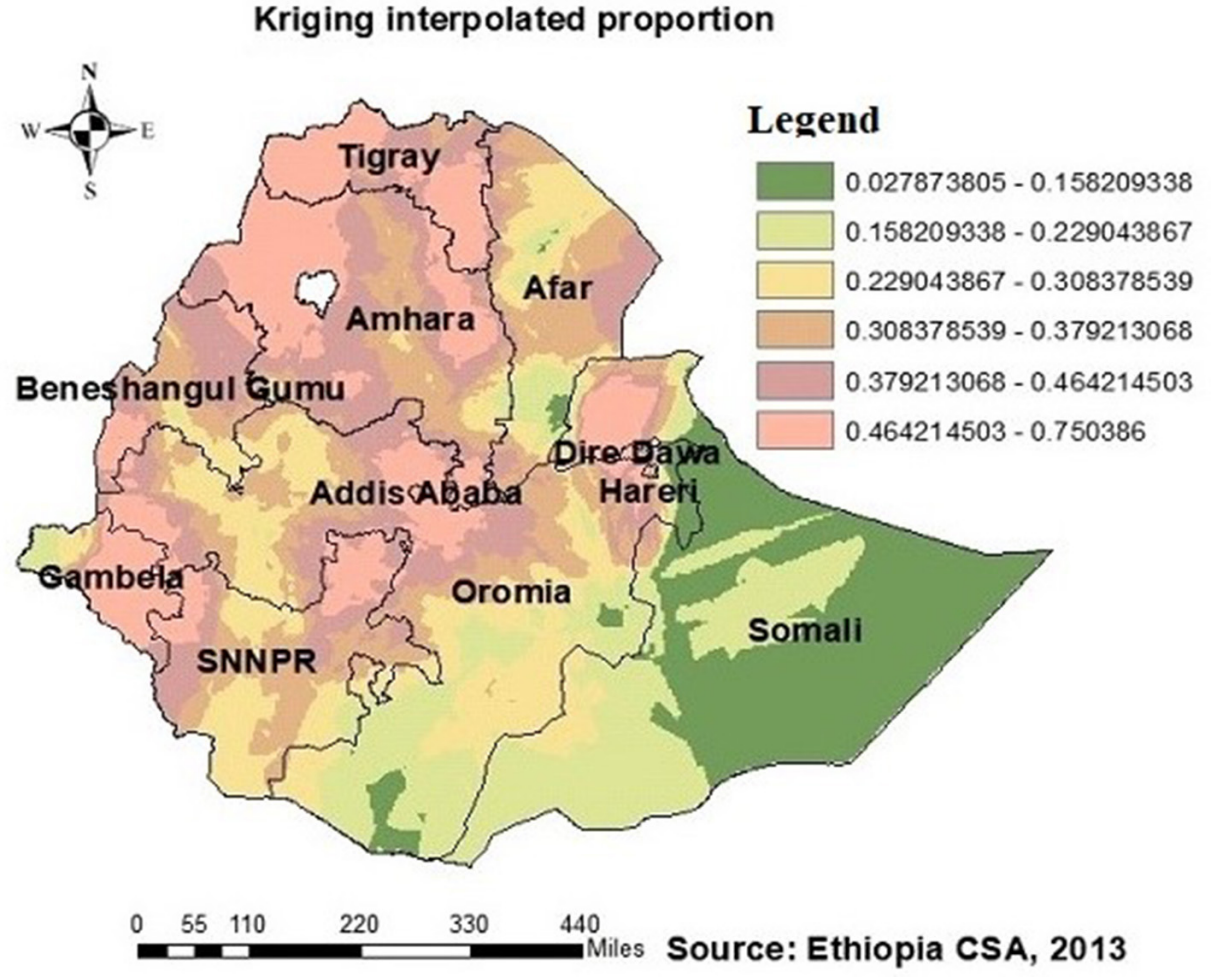
HIV ever-tested AGYW in Ethiopia using kriging interpolation. The interpolated areas (orange) were with high proportion of AGYW ever-tested for HIV while interpolated areas (olive green) were with low proportion of AGYW ever-tested for HIV.

Regarding the autocorrelation analysis, findings showed that significant spatial variance on being ever-tested for HIV with Global Moran's I was 0.46 and *p*-value was 0.000 (Figure 2). Addis Ababa, the northern part of Amahar, Dire Dawa, Gambela, and Tigray regions were shown to be the statistically significant hotspot areas for being ever-tested for HIV. In contrast, Afar, most parts of Amhara, Benshangul Gumuz, SNNPR, and Somalia were significant cold spots (Figures 3, 4).

### Predictors of ever tested for HIV

The OLS model identifies the relationships between independent and dependent variables based on geographic locations and affects HIV ever-tested in AGYW in Ethiopia with an adjusted *R*^2^ of 0.758; this model accounted for 75.8% of the variation in being ever-tested for HIV. If the Koenker BP test in this study showed a significant association, the geographically weighted regression is advised. The Jarque–Bera statistic was not significant (*P* = 0.060); hence, it was believed that the model residuals would be normally distributed. In addition, the Joint Wald statistic demonstrated that the entire model was significant (*P* < 0.001) (Table 2). Since each explanatory variable had a local coefficient, we used a geographically weighted regression model and retrieved those values. The study identified various factors that predict the locations of hotspots for HIV testing, including the percentage of adolescent girls and young women (AGYW) aged 15–19 years, the proportion of Muslim individuals, the proportion of those with no formal education, the proportion of those not knowledgeable about HIV, and the proportion of community stigma (Table 2). The proportion of being ever-tested for HIV differed among AGYW in various demographic groups. For AGYW aged 15–19 years, with no formal education, not knowledgeable about HIV, and experiencing community stigma, the likelihood of being ever-tested for HIV decreased by −0.30, −0.21, 0.22, and −0.22 times, respectively. Conversely, the proportion of Muslim AGYW who reported being ever-tested for HIV increased by 0.16 times with an increase in their proportion.

**Table 2:** Predictors of a high proportion of ever been tested for HIV: result from secondary data analysis of EDHS 2016.

**Variable**	**OLS-coefficient**	**Standard error**	***t*-statistic**	**Probability**	**Robust standard error**	**Robust *t*- statistics**	**Robust probability**	**VIF**
Age:15–19 years	−0.30	0.04	−7.6	0.000	0.04	−6.8	0.000	1.1
Muslim	0.16	0.05	2.26	0.024	0.03	2.0	0.045	2.3
No formal education	−0.21	0.03	−4.9	0.000	0.04	−4.6	0.000	2.5
No knowledge about HIV	−0.22	0.04	−5.3	0.000	0.04	−4.7	0.000	2.7
Severe stigma	−0.22	0.04	5.1	0.000	0.05	−4.8	0.000	2.7

### Geographically weighted regression analysis

**Figure 5:**
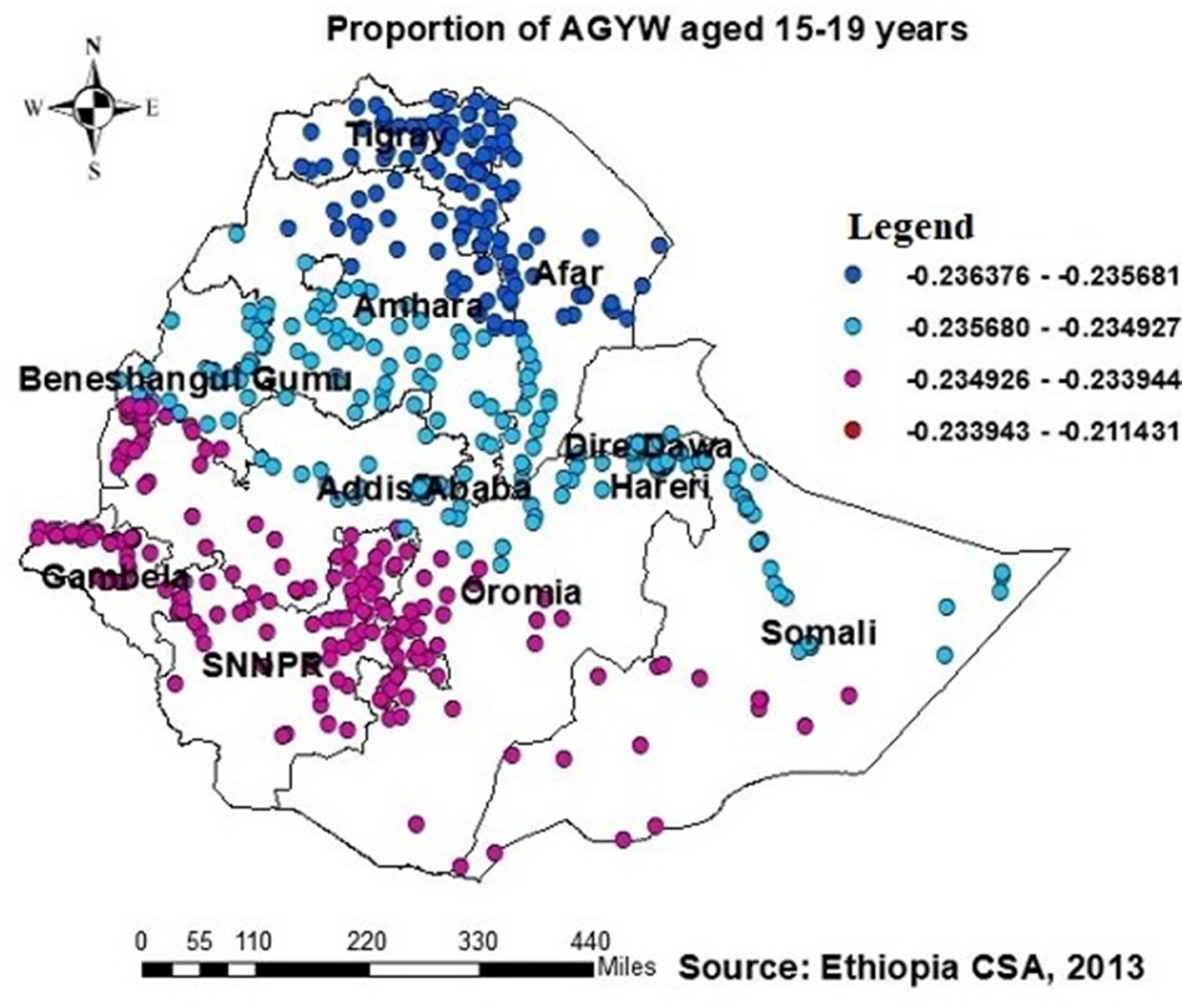
GWR coefficients of the proportion of AGYW aged 15–19 years as a predictor of those being ever-tested for HIV. The proportion of ever-tested for HIV (b1ue legend areas) increases when the proportion of adolescent girls and young women (AGYW) aged 15–19 years increases, while the proportion of ever-tested for HIV (red legend areas) decreases when the proportion of AGYW increases.

The GWR analysis showed an improvement in the model. The findings indicated a notable difference in the “AICc” values, with a decrease from −316.3 in the OLS regression to −329.13 in the GWR analysis (Table 3). Moreover, the adjusted *R*^2^ value of 0.758 obtained using ordinary least squares (OLS) regression increased to 0.78 by use of GWR. This finding indicates that GWR improved the model's ability to predict the likelihood of individuals having been tested for HIV. Therefore, the results of this research indicate that the GWR analysis showed superior performance compared to the OLS model (Table 3). The model identified both positive and negative associations with the proportion of being ever-tested for HIV. The proportion of being ever-tested for HIV increases with an increase in the proportion of AGYW aged 15–19 years in Tigray, central Afar, Amhara, Addis Ababa, Dire Dawa, Gambela, Harari, and a central part of the Oromia region (Figure 5). However, the proportion of being ever-tested for HIV decreased in Gambela (south and southwest) Oromia, SNNPR, and (south and western) Somalia.

**Table 3:** Diagnosis of regression of ordinary least square (OLS).

**Number of observations**	**643**	**AICc**	**−316.3**
Multiple *R*^2^	0.76	Adjusted *R*^2^	0.758
Joint *F*-statistic	68.8	Prob(>F), (10,632) degrees of freedom	0.000
Joint Wald statistic	1,263.2	Prob(>chi-squared), (10) degrees of freedom	0.000
Koenker (BP) statistic	56.6	Prob(>chi-squared), (10) degrees of freedom	0.000
Jarque–Bera statistic	6.8	Prob(>chi-squared), (2) degrees of freedom	0.56

**Figure 6:**
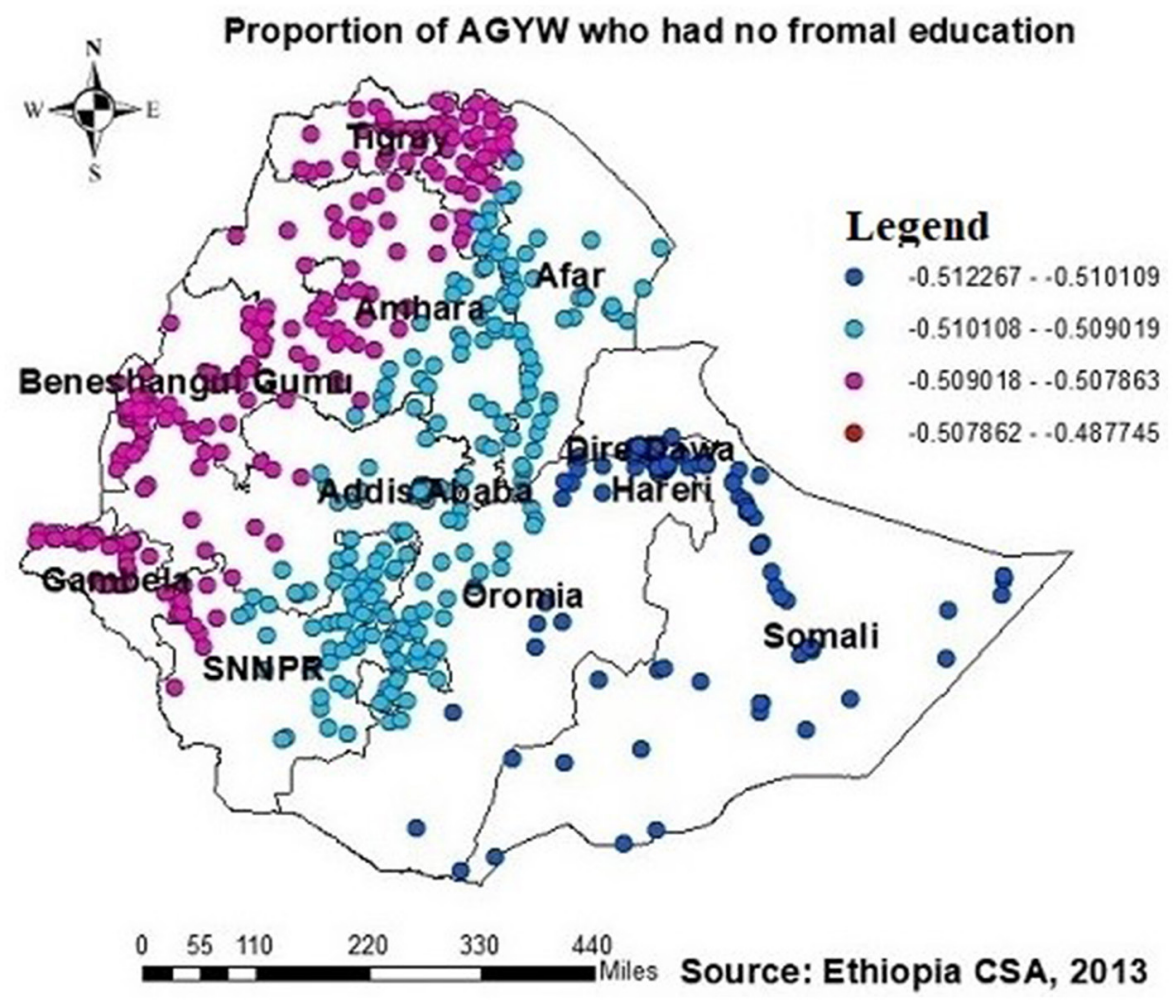
GWR coefficients of the proportion of those with no formal education as a predictor of those being ever-tested for HIV. The proportion of ever-tested for HIV (b1ue legend areas) decreases when the proportion of no formal education increases, while the proportion of ever-tested for HIV (red legend areas) increases when the proportion of no formal education decreases.

**Figure 7:**
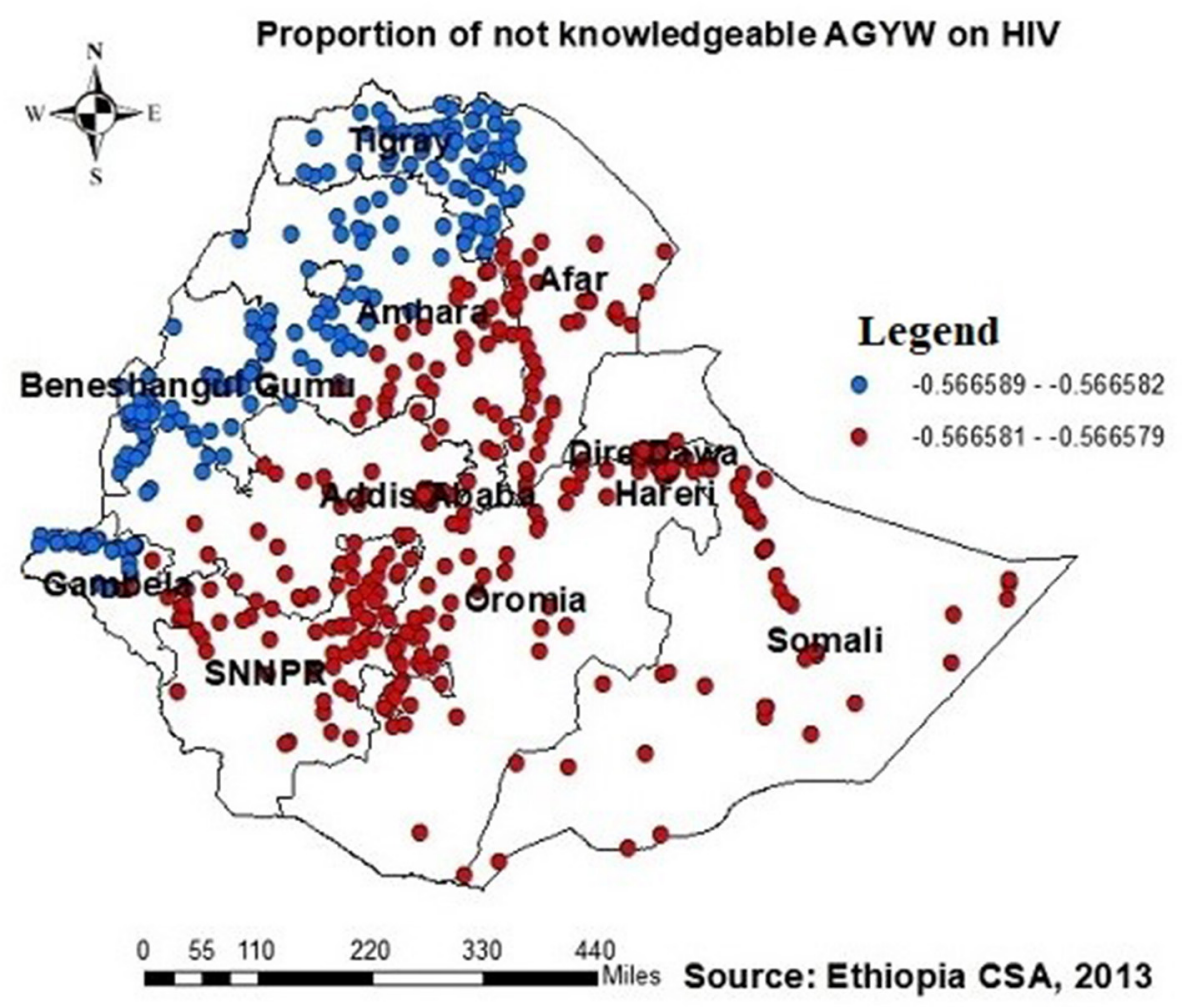
GWR coefficients of the proportion of those not knowledgeable about HIV as a predictor of those being ever-tested for HIV. The proportion of ever-tested for HIV decreases (b1ue legend areas) decreases when the proportion of those not knowledgeable about HIV increases, while the proportion of ever-tested for HIV (red legend areas) increases when the proportion of those not knowledgeable about HIV decreases.

**Figure 8:**
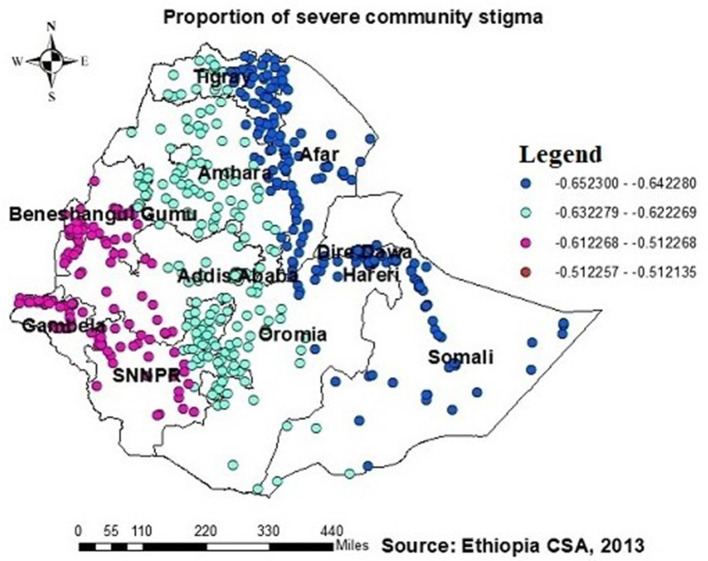
GWR coefficients of the proportion of those experiencing severe community stigma as a predictor of those being ever-tested for HIV. The proportion of ever-tested for HIV (b1ue legend areas) decreased when community stigma was identified as a prominent contributing factor.

The proportion of individuals with no formal education showed a negative relationship with the proportion of those being ever-tested for HIV. The proportion of individuals being ever-tested for HIV decreased in Afar, Addis Ababa, southern Amhara, Dire Daw, Harari, most of the Oromia region, Somalia, and SNNPR with an increase in the proportion of no formal education (Figure 6). Regarding knowledge of HIV, the proportion of those not knowledgeable about HIV showed a negative association with the proportion of those being ever-tested for HIV. The proportion of individuals being ever-tested for HIV decreased in the northern areas of Amhara, Gambela, Benshangul Gumezu, and Tigra with an increase in the proportion of those not knowledgeable about HIV (Figure 7). The research finding showed a negative correlation between the proportion of community stigma and the proportion of those being ever-tested for HIV. The areas of Afar, Addis Ababa, Harari, Amhara, and the northern part of SNNPR, Oromiya, and Tigray had a decreased proportion of those being ever-tested for HIV when community stigma was identified as a prominent contributing factor (Figure 8).

**Figure 9:**
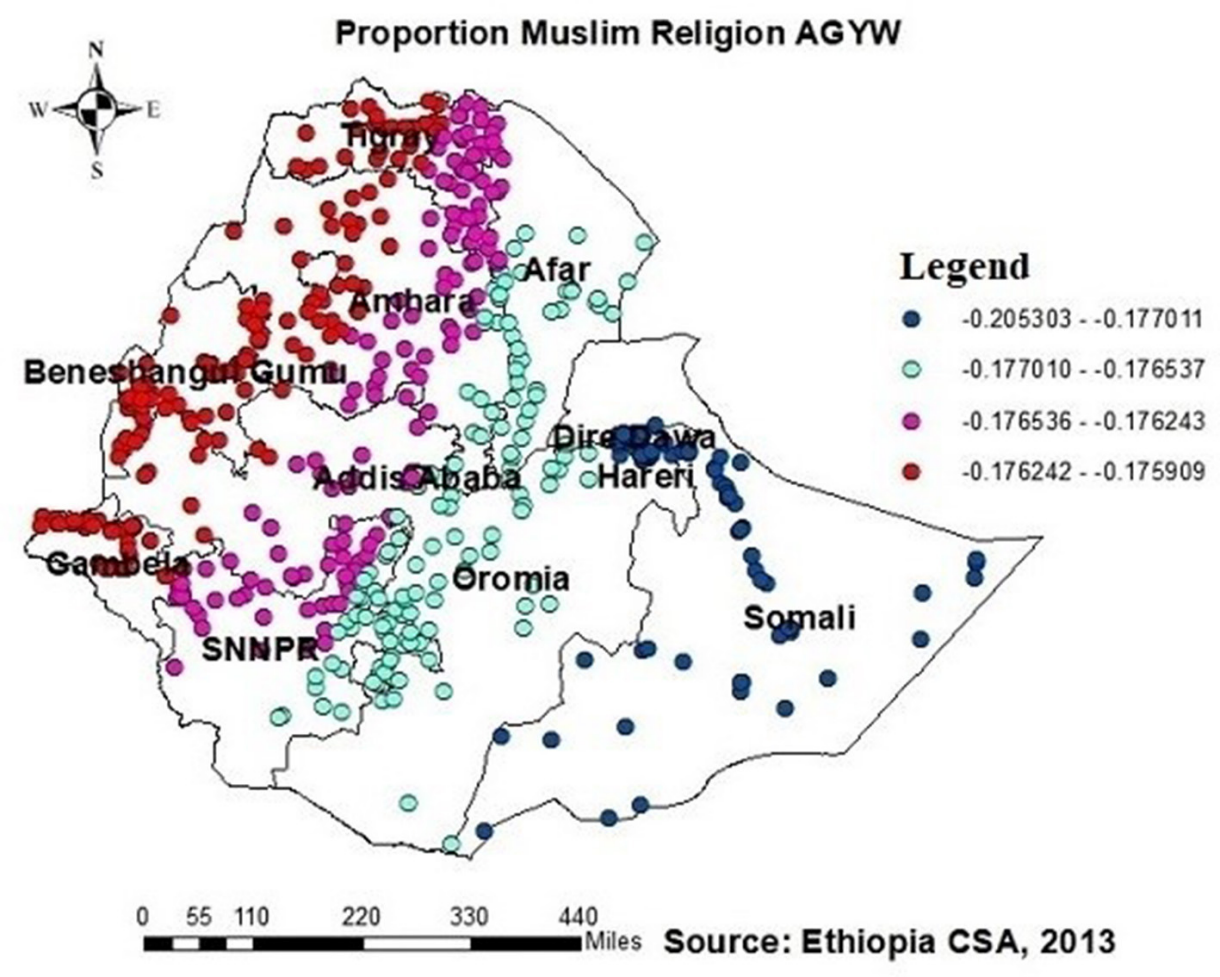
GWR coefficients of the proportion of AGYW being Muslim as a predictor of those being ever-tested for HIV. The proportion of ever-tested for HIV (b1ue legend areas) increases when the proportion of Muslim AGYW increases, while the proportion of ever-tested for HIV (red legend areas) less increases when the proportion of Muslim AGYW increases.

In Addis Ababa, Afar, the southern part of Amhara, Dire Dawa, the northern part of SNNPR, and (south and southeast) part of the Oromia region, Harari, and Somalia, the proportion of muslims had a significant and positive association with the proportion of those being ever-tested for HIV (Figure 9).

## Discussion

The study identified hotspots of being ever-tested for HIV among AGYW in Addis Ababa, northern Amhara, Dire Dawa, Tigray, and Gambela regions. Cold spot regions, such as Afar, most of Amhara, Somalia, Benishangul-Gumuz, and SNNPR, were also identified. These findings align with those of previous studies conducted on the spatial distribution of HIV testing and counseling in Ethiopia (18, 21), as well as a similar study in Nigeria. This finding is supported by the geographic concentration of HIV infections within the nation and regional disparities in prioritizing HIV testing (22, 31–33). The presence of cold spots in HIV testing could be linked to the limited availability of medical services in specific areas. Conversely, hotspot regions, which are often characterized by higher levels of economic development and infrastructure, tend to have better access to healthcare facilities and HIV testing services, leading to higher testing rates. On the contrary, cold spot regions may face economic challenges or political instability, making it difficult to provide sufficient healthcare services, thereby resulting in lower testing rates.

Furthermore, the differences in education access across geographical regions, media exposure, cultural factors, and living conditions may contribute to these variations (21). For example, our study revealed that 54.97% of AGYW in Somalia lacked formal education, whereas only 3.9% of AGYW in Addis Ababa had no formal education. Although the proportion of media exposure did not exhibit a direct relationship with the high prevalence of HIV testing in certain areas, our findings indicate that only 2.4% of AGYW in Addis Ababa lacked media exposure, while a significant 74.69% of AGYW in Somalia were not exposed to media. This lack of media exposure in Somalia likely contributed to lower rates of HIV testing than in other regional states in Ethiopia, consistent with findings from other studies (34, 35). While the use of media platforms such as radio, television, and social media can effectively increase HIV awareness and encourage HIV testing, depending solely on these media for disseminating health information may marginalize individuals with restricted access to electronic media, especially in rural or isolated areas where such platforms are scarce.

This study employed GWR models to analyze the spatial variations and predictors of the proportion of HIV testing among AGYW in Ethiopia. Positive and negative associations with the proportion of those being ever-tested for HIV were identified, including the proportion of participants aged 15–19 years, who lack of formal education, being Muslim, who lack of knowledge about HIV, and who are experiencing community stigma.

The current study found a negative association between the proportion of AGYW aged 15–19 and those prediciting being ever-tested for HIV. The GWR coefficients ranged from 0.23 to 0.21 across districts, indicating a negative association with HIV testing among AGYW. This finding suggests a lower uptake of HIV testing among AGYW aged 15–19. Similar findings are reported in other studies (21, 23, 34). This can be attributed to the decreased self-perceived risk of HIV among AGYW aged 15–19 (21, 36, 37). Additionally, factors such as lower engagement in sexual activity, delayed marriage, and limited economic power among AGYW aged 15–19 contributed to this trend (38).

The study revealed a significant negative association between the proportion of AGYW with no formal education and those being ever-tested for HIV. The GWR coefficients for the proportion of AGYW with no formal education ranged from 0.51 to 0.48. This factor strongly influenced the likelihood of them being ever-tested for HIV, with notable occurrences in regions such as Afar, Addis Ababa, southern Amhara, Dire Dawa, Harari, most of the Oromia region, Somalia, and SNNPR.

Educated AGYW are more likely to understand the benefits of HIV testing and the importance of protection against HIV infection through various media sources such as newspapers, television, and social media, as evidenced in studies from Burkina Faso, Ethiopia, Malawi, Nigeria, and Zambia (21, 34, 35, 39–42). Conversely, lack of formal education constrains access to information and awareness about the importance of HIV testing. Additionally, it diminishes one's autonomy in making decisions to seek healthcare services and utilize healthcare facilities (11, 21, 43, 44).

Similarly, the proportion of individuals lacking knowledge about HIV showed a negative relationship with the proportion of those being ever-tested for HIV, with GWR coefficients ranging between 0.57 and 0.56. This finding is supported by disparities across geographical regions in awareness of HIV transmission and the importance of HIV testing in Ethiopia (45), leading to a varied distribution of being ever-tested AGYW across the country. Moreover, communities with limited knowledge about HIV may experience increased HIV-related stigma, resulting in reduced rates of HIV testing due to decreased awareness (11, 21, 46, 47).

Additionally, this study revealed a negative relationship between the proportion of individuals being ever-tested for HIV among AGYW and severe community stigma. The GWR coefficients for the proportion of severe community stigma ranged between 0.65 and 0.51 (Figure 8). This finding is consistent with those of studies conducted in Ethiopia, China, Malawi, and Nigeria (21, 23, 34, 37, 39, 42, 48). However, in regions such as Tigray, Amhara, Dire Dawa, the northern part of southern Ethiopia, Gambela, and southwest and southern Shewa Oromia, the proportion of muslim AGYW exhibited a positive association with those being ever-tested for HIV, with GWR coefficients ranging from 0.21 to 0.17. This finding may be attributed to muslim participants potentially having better access to education than orthodox and other religious groups; while it is possible for muslim communities to arrange health-related initiatives during religious gatherings or prayer days, these endeavors must prioritize HIV testing and ensure accessibility to the broader community to have a notable effect. Nonetheless, there is limited evidence indicating that such endeavors are prevalent or unique to muslim communities.

Several studies conducted in various Sub-Saharan African countries have highlighted the significance of access to healthcare facilities for HIV testing (19, 49–52). However, in this study, the proportion of distance to healthcare facilities did not show a significant relationship with the proportion of those being ever-tested for HIV among AGYW. Similar findings have been reported in other studies conducted in Ethiopia (21, 53). This could be due to AGYW traveling long distances to access healthcare services to avoid community stigma. Studies conducted in South Africa have also shown that individuals often travel significant distances to access healthcare facilities, indicating that individuals living near regional borders might access services outside their residential region (54, 55). This finding suggests that healthcare access patterns might not strictly adhere to regional boundaries.

The current analysis comprised a representative population of AGYW in Ethiopia. In addition, most of the previous studies identified factors using the logistic regression analysis. However, this study used OLS and GWR to account for geographical or regional heterogeneity variation in determining the factors of high HIV ever-tested areas. Despite the strengths mentioned above, the study has its limitations. The original study was a cross-sectional survey, causing difficulty in establishing a cause and relationship. In addition, self-response indicated that there could be potential memory loss among AGYW, and the original study might cause socially desirable biases. Finally, due to the secondary data used in this study, crucial variables such as service delivery, including quality, cost, distance, and accessibility to HIV testing services, were not assessed in this study, which could cause missing important predictors.

## Conclusion

The proportion of hotspot regions for HIV ever-tested for HIV hotspot regions found include Amhara, Addis Ababa, Dire Dawa, Tigray, and Gambela regions. Somali, Afar, Benshangul Gumuz, and SNNPR regions were demarcated as cold spot regions for HIV ever-tested. AGYW aged 15–19 years, Muslim religion, had no formal schooling, no knowledge on HIV, and with severe community stigma were found to be predictors of HIV ever-tested in hotspot areas using GWR. To prevent HIV infection and control the disease, governments and other stakeholders should give due attention to increasing HIV testing among these special groups of the population.

## Data availability statement

The data analyzed in this study is subject to the following licenses/restrictions: it is EDHS data that will be available to the corresponding author and will be given up on request. Requests to access these datasets should be directed to DHS; email address: archive@dhsprogram.com; web: https://dhsprogram.com/.

## Ethics statement

Ethical approval was not required for the study involving humans in accordance with the local legislation and institutional requirements. Written informed consent to participate in this study was not required from the participants or the participants' legal guardians/next of kin in accordance with the national legislation and the institutional requirements.

## Author contributions

MS: Data curation, Formal analysis, Methodology, Project administration, Resources, Software, Supervision, Validation, Visualization, Conceptualization, Writing – review & editing, Writing – original draft. AT: Conceptualization, Data curation, Formal analysis, Methodology, Project administration, Resources, Software, Supervision, Validation, Visualization, Writing – review & editing, Investigation. BB: Writing – review & editing, Visualization, Validation, Supervision, Resources, Project administration, Methodology, Formal analysis, Data curation. AG: Conceptualization, Data curation, Writing – review & editing, Writing – original draft, Visualization, Validation, Supervision, Software, Resources, Project administration, Methodology, Investigation, Formal analysis. SH: Conceptualization, Data curation, Formal analysis, Investigation, Methodology, Resources, Software, Supervision, Validation, Writing – review & editing. LF: Writing – review & editing, Visualization, Software, Methodology, Investigation, Conceptualization. WM: Conceptualization, Data curation, Formal analysis, Investigation, Methodology, Project administration, Resources, Software, Supervision, Validation, Visualization, Writing – original draft, Writing – review & editing.
